# Special Issue: “Pelvic Floor Disorders: State of the Art and Future Perspectives”

**DOI:** 10.3390/jcm11216473

**Published:** 2022-10-31

**Authors:** Barbara Bodner-Adler

**Affiliations:** Department of General Gynecology & Gynecologic Oncology, Medical University of Vienna, Währinger Gürtel 18-20, 1090 Vienna, Austria; barbara.bodner-adler@meduniwien.ac.at

This editorial for the Special Issue “Pelvic Floor Disorders: State of the Art and Future Perspectives” aims to draw attention to the broad field of pelvic floor disorders and serves as an invitation for researchers on a global scaleto share their most recent findings with the urogynecologic community. Pelvic floor disorders (PFDs) such as urinary incontinence (UI) and pelvic organ prolapse (POP) are common diseases which can significantly affect women’s quality of life.

**Pelvic organ prolapse (POP):** Pelvic organ prolapse (POP) is a major health issue worldwide, affecting up to 50% of postmenopausal women. Treatment options for POP include observation, pelvic floor muscle training, pessary use and pelvic floor surgery. Surgical solutions considered “one-off” treatments, rather than nonsurgical treatments, were found to be preferred by women with POP [1]. Furthermore, surgery for POP currently has a lifetime risk of 11.1%, and due to increasing age, is expected to increase in the future [2]. Therefore, we expect several challenges regarding pelvic floor surgery in the future. Schulten et al., for example, performed a randomized controlled trial (RCT) on 204 female patients with POP. The patients were randomized and either underwent with prolapse hysterectomy and apical fixation, or they underwent uterus-preserving sacrospinous hysteropexy as an alternative to vaginal hysterectomy. The results showed significantly diminished rates of recurrence and repeat surgery, as well as a lower morbidity, in the group with uterus preservation after 5 years [3]. 

Such publications emphasize that the “one-size-fits-all“ strategy has ceased to be an acceptable solution and personalized treatment offers will dominate in the future. Furthermore, molecular studies investigating the muscular and connective tissue of patients with POP will be an important research topic in the future. It is known that the pelvic organs, as well as the surrounding muscular and connective tissue, are sensitive to estrogen. The presence of estradiol receptors a and b (ESR1/2) in the urinary bladder, urethra, vagina and pelvic floor musculature suggest that estrogen levels have a significant effect on the function of the genitals and lower urinary tract [4,5]. The risk of developing any pelvic floor disorder increases significantly after menopause and could be connected to the decline in available estrogen. 

**Urinary incontinence:** Overactive bladder (OAB) syndrome and stress urinary incontinence (SUI), are also common diseases. Their treatment involves conservative approaches such as weight reduction or improved fluid intake, as well as physical therapy in the form of pelvic floor or bladder training. Their pharmacological medications include antimuscarinic and beta-adrenergic drugs, as well as estrogen agents. Additionally, incontinence surgery in cases of SUI is a further option. Additionally, OAB is a socio-economic burden incurring high annual healthcare costs, and also has a social impact on affected women, who may experience social isolation, loss of confidence and depression [6,7]. The exact causes and pathophysiologic mechanisms of OAB are still poorly understood, leaving patients with less-than-optimal treatment options. Neuronal, myogenic and inflammatory processes have previously been discussed regarding their influence on OAB, with limited results [8]. 

The above-mentioned literature review highlights the complex role of pelvic floor disorders and their significant increase with age in women, as well as their significant impact on women’s quality of life. This Special Issue aims to further improve our understanding of preventing, controlling and treating pelvic floor disorders in affected women. 
